# Target controllability: a feed-forward greedy algorithm in complex networks, meeting Kalman’s rank condition

**DOI:** 10.1093/bioinformatics/btae630

**Published:** 2024-10-23

**Authors:** Seyedeh Fatemeh Khezri, Ali Ebrahimi, Changiz Eslahchi

**Affiliations:** Department of Computer and Data Sciences, Shahid Beheshti University, Tehran 1983969411, Iran; School of Biological Sciences, Institute for Research in Fundamental Sciences (IPM), Tehran 19395-5746, Iran; Department of Computer and Data Sciences, Shahid Beheshti University, Tehran 1983969411, Iran; School of Biological Sciences, Institute for Research in Fundamental Sciences (IPM), Tehran 19395-5746, Iran

## Abstract

**Motivation:**

The concept of controllability within complex networks is pivotal in determining the minimal set of driver vertices required for the exertion of external signals, thereby enabling control over the entire network’s vertices. Target controllability further refines this concept by focusing on a subset of vertices within the network as the specific targets for control, both of which are known to be NP-hard problems. Crucially, the effectiveness of the driver set in achieving control of the network is contingent upon satisfying a specific rank condition, as introduced by Kalman. On the other hand, structural controllability provides a complementary approach to understanding network control, emphasizing the identification of driver vertices based on the network’s structural properties. However, in structural controllability approaches, the Kalman condition may not always be satisfied.

**Results:**

In this study, we address the challenge of target controllability by proposing a feed-forward greedy algorithm designed to efficiently handle large networks while meeting the Kalman controllability rank condition. We further enhance our method’s efficacy by integrating it with Barabasi et al.’s structural controllability approach. This integration allows for a more comprehensive control strategy, leveraging both the dynamical requirements specified by Kalman’s rank condition and the structural properties of the network. Empirical evaluation across various network topologies demonstrates the superior performance of our algorithms compared to existing methods, consistently requiring fewer driver vertices for effective control. Additionally, our method’s application to protein–protein interaction networks associated with breast cancer reveals potential drug repurposing candidates, underscoring its biomedical relevance. This study highlights the importance of addressing both structural and dynamical aspects of network controllability for advancing control strategies in complex systems.

**Availability and implementation:**

The source code is available for free at:Https://github.com/fatemeKhezry/targetControllability.

## 1 Introduction

In recent years, one of the important challenges in the field of network science has been the feasibility of controlling complex networks, where the desired system is represented by a set of vertices, and the connections between them are manifested as network edges. In this context, the system is not limited to small technical systems and can contain thousands of variables. Control theory aims to find appropriate input signals for the system, which can, based on the connections between its components, change the state of all variables from unfavorable to favorable in finite time (Kalman 1963, Slotine and Li 1991). Neural networks, metabolic networks, gene expression regulatory networks, protein–protein interaction (PPI) networks, etc., are among the biological systems that are usually complex and large. Controlling their vertices to change their state from an undesirable condition (e.g. illness) to a desired one (e.g. health) is a critical goal, and many applications have been proposed for such changes (Liu and Pan 2014, Wuchty 2014, Wu *et al*. 2015, 2017, Li *et al*. 2019).

It is straightforward to control systems with a few variables where applying control signals to all the network’s vertices is feasible. However, as the number of network vertices increases, it becomes impractical to apply external signals to all vertices for network control. Therefore, identifying the smallest number of vertices in the network that, when affected by external signals, can control the entire network (known as controllability) or a subset of the network that needs to be controlled (known as target controllability) is of great interest to researchers. For instance, network control methods are used to identify a small set of drug targets in biomolecular networks. In this view, drug targets act as driver vertices to control the network. This implies that when driver vertices are influenced by external signals, such as drugs or other treatments, they can systematically influence all vertices within the network, or specifically those vertices associated with a particular disease that have been designated as control targets (Asgari *et al*. 2013, Setyawan *et al*. 2019, Guo *et al*. 2021).

Understanding the controllability of complex networks is paramount for effectively manipulating their behavior, particularly in dynamical systems. By identifying key driver vertices and understanding how external inputs can influence the entire network or specific target vertices, we gain valuable insights into network behavior and control strategies. This understanding becomes especially important when considering real-world systems, where nonlinear dynamics are prevalent. Despite the inherent complexity of these systems, controllability concepts often extend from linear to nonlinear systems, facilitating analysis and control. Thus, while real dynamical systems may exhibit nonlinear behavior, for simplicity and analytical tractability, they are often modeled as linear time-invariant (LTI) systems (Kalman 1963, Yuan *et al*. 2013). Following this understanding, the evolution of a linear time-invariant system over time can be precisely described by an equation governing its dynamics as:

(1) $$\{\begin{array}{c} \frac{dx(t)}{dt}=Ax(t)+Bu(t)\\ y=Cx. \end{array}$$

where $x\in R^{n},\;u\in R^{p}$, $y\in R^{m}$, $\;A\in R^{n\times n},\;B\in R^{n\times p}$, and $C\in R^{m\times n}$ represent the system’s state, input, and output vector, and the state, input, and output matrices, respectively.

An LTI system given in Equation (1) can be seen as a graph *G*(*V*, *E*), where *V* and *E* denote the set of vertices and the set of edges, respectively. In this context, the state vector $x(t)=(x_{0}(t),x_{1}(t),\ldots ,x_{n-1}(t))$ denotes the state or condition associated with each vertex at time *t*, and the matrix *A* represents the transpose of the adjacency matrix of the graph *G*. The element *a_ij_* represents the weight of the directed edge from *v_j_* to *v_i_*. If $D=\{d_{0},d_{1},\ldots ,d_{p-1}\}\subset V$ are the input vertices, the matrix *B* is defined as follows:

(2) $$B_{D}=[I(d_{0}),I(d_{1}),\ldots ,I(d_{p-1})]$$

where $I(d_{i})$ denotes the *i*th column of the identity matrix of size *n*. In target controllability to control a target vertices $T=\{c_{0},c_{1},\ldots ,c_{m-1}\}$, the matrix *C* is defined as follow:

(3) $$C_{T}=[I(c_{0});I(c_{1});\ldots ;I(c_{m-1})]$$

where $I(c_{i})$ denotes the *i*th row of the identity matrix of size n. In exact controllability, Kalman introduced a condition for LTI systems (Kalman 1963). According to this condition, a system described by Equation (1) is controllable by set D if and only if the control matrix $M(A,B_{D})$ introduced in Equation (4) has full rank:

(4) $$M(A,B_{D})=[B_{D}|AB_{D}|A^{2}B_{D}|\ldots |A^{n-1}B_{D}]$$

When the aim is to control the set of vertices *T* (indicates the target vertexs in *G*) by *D*, the condition is rewritten to say that the system described by Equation (1) is target controllable by set D if and only if the control matrix $CM(A,B_{D},C_{T})$ introduced in Equation (5) has full rank (Murota and Poljak 1990):

(5) $$CM(A,B_{D},C_{T})=[C_{T}B_{D}|C_{T}AB_{D}|C_{T}A^{2}B_{D}|\ldots |C_{T}A^{n-1}B_{D}]$$

Structural controllability, introduced by Lin, provides a more tractable condition for analyzing the controllability of complex networks compared to Kalman’s condition. In structural controllability, only directed networks with structural matrices are considered, where the presence or absence of edges is considered, and any additional parameters specifying the weight of the edges are ignored (Lin 1974). In this context, Barabasi et al. introduced the minimum input theorem, which is centered on understanding how to control complex networks by employing a concept called “maximum matching.” Unmatched vertices are identified as “driver” vertices. This theorem offers a method to pinpoint these driver vertices, providing valuable insights for managing large and intricate networks. Ultimately, it aids in identifying the smallest set of vertices required to effectively control the network’s behavior (Liu *et al*. 2011).

Target controllability, which seeks to control a specific target in the network instead of its full control, is also a necessary and useful challenge. The findings for full control of the network, both in exact control and structural control, reveal that in large networks, achieving controllability with a small number of inputs is not feasible. However, in various networks, such as biological networks, there is often no need to control all vertices within the network. Instead, the focus can be on controlling specific portions of the network that are crucial for achieving the desired target (Gao *et al*. 2014). On the other hand, target controllability is an NP-hard problem (Czeizler *et al*. 2016). To tackle this complexity, various solutions have been proposed. It is evident that by considering all network vertices as the control target, any target controllability algorithm can also check the full controllability of the network. One notable approach is the application of multiple maximum matches, which involves a greedy algorithm based on the minimum input theorem (BAGA Algorithm) (Gao *et al*. 2014). GeneticAlg is also one of the well-known target controllability methods that determine the set of driver vertices based on the genetic algorithm (Popescu *et al*. 2022). Another considered algorithm is the TCMM, a target controllability method with minimal mediation based on the path length of pairs of vertices in the network (Ebrahimi *et al*. 2020b). Additionally, several other algorithms have been developed to identify the minimum number of driver vertices required to control the desired target within the network (Monshizadeh *et al*. 2015, Czeizler *et al*. 2016, Czeizler *et al*. 2018, Ebrahimi *et al*. 2020b, Li *et al*. 2020, Hao *et al*. 2022). Significant works such as Xue and Bogdan (2019), Ghorbani *et al*. (2018), Kyriakis *et al*. (2020), Reed *et al*. (2023), Ebrahimi *et al*. (2020a), Gupta *et al*. (2018), and Yang and Bogdan (2020) have been done on gene regulatory networks, long-term power-law memory, and the effects of memory and topology on the controllability of complex dynamical networks, which provide crucial insights into the behavior of biological systems. These studies emphasize the need for control strategies that consider the unique characteristics of biological networks, such as their fractal nature and long-range dependencies.

In this article, we introduce a novel algorithm called the Greedy Target Controllability Feed-forward Algorithm (GTCA), developed to tackle the problem of target controllability. The “feed-forward” aspect refers to its unidirectional processing of inputs through the network, without the use of feedback loops, which ensures efficient and accurate selection of driver vertices. GTCA stands out by meeting the stringent Kalman condition, ensuring that the driver vertices identified can effectively control the desired target. Furthermore, our approach demonstrates robust performance even in large and complex networks, showcasing its scalability and applicability across diverse scenarios. We have extensively tested our method on various biological networks, including gene networks, signaling pathways, and PPI networks. Our case study focuses specifically on protein networks related to breast cancer. In the remaining sections of the article, we present a comprehensive overview of our proposed method. Each step of the algorithm is elucidated in detail, providing readers with a clear understanding of its workings and implementation. Subsequently, we conduct an extensive evaluation of GTCA’s performance by comparing its results with those obtained from several state-of-the-art methods. These comparisons are carried out across various datasets to thoroughly assess the efficacy and robustness of the presented method. Additionally, in the case study section, we investigate the biological significance of the driver vertices identified by the new algorithm in breast cancer PPI networks.

## 2 Materials and methods

**Figure 1.**
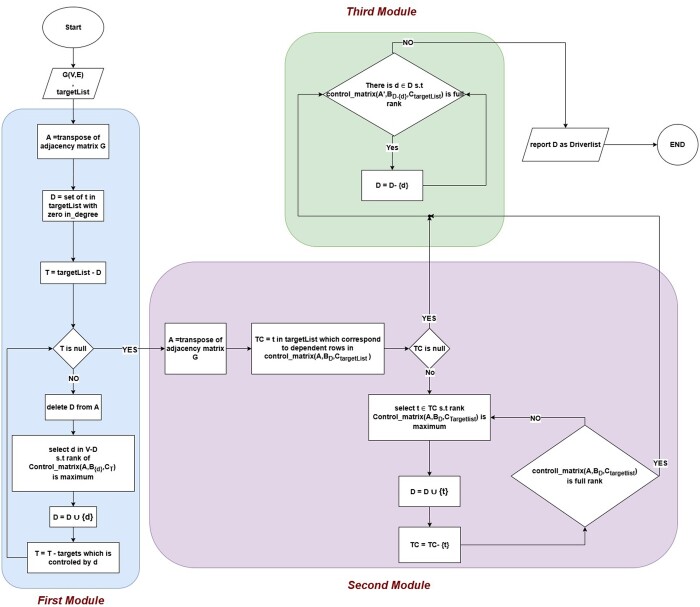
Flowchart of the proposed target controllability algorithm.

For any arbitrary graph *G* and any control target set *T*, the GTCA comprises three modules, each designed to identify the set of driver vertices (Fig. 1).

**Module 1:** The first module identifies a subset of vertices as the initial set of primary input vertices, or drivers. Each vertex in this subset individually satisfies Kalman’s rank-controllability condition for a fraction of the target set T. However, this subset may not necessarily satisfy Kalman’s rank-controllability condition for the entire target set.

**Module 2:** In the second module, additional vertices from the target set are added to the drivers set. This process continues until the expanded drivers set satisfies Kalman’s rank-controllability condition for the entire target set.

**Module 3:** In the third module, the algorithm refines the drivers set by removing any candidate drivers that do not impact the rank of the control matrix. The goal is to minimize the driver set while ensuring that Kalman’s condition remains satisfied.

In the following, the steps of the GTCA method are explained in detail:

### 2.1 Algorithm

Let G(V,E) be a directed weighted graph with n vertices where $V=\{v_{0},v_{1},\ldots ,v_{n-1}\}$ be the set of vertices, and $A_{n\times n}$ be the transpose of adjacency matrix of G. Suppose $T=\{t_{0},t_{1},\ldots ,t_{m-1}\}$ is a given target set. By the following steps, the algorithm finds a subset of V like $D=\{d_{0},d_{1},\ldots ,d_{p-1}\}$ to control *T* such that the size of *D* is minimal.

*Part one of the algorithm:*

1. Define D=∅
2. Find all the vertices in the targets set which have in_degree = 0 in graph G and add them to set *D*.
3. If D=∅ go to step 9. Otherwise:
4. Let $CM(A,B_{D},C_{T})$ be the control matrix which is defined in equation 4 that *B_D_* and *C_T_* are the matrices which are defined in equations 2 and 3 respectively.
5. Find the maximum set of independents rows of the $CM(A,B_{D},C_{T})$. Each row correspond to one controlled target vertex by set *D*. Let *S* be the set of all vertices that are controlled by *D*.
6. Let T=T−S be the uncontrolled targets.
7. Remove all rows and columns corresponding to the vertices in the set *D* from *A*. We rename the new matrix as *A* again.
8. If T=∅, go to part two of algorithm. Otherwise:
9. Calculate $C_{T}A,C_{T}A^{2},C_{T}A^{3},\ldots ,C_{T}A^{n-1}$ which the matrix *A^i^* is the i-th power of *A*.
10. Calculate the rank of each matrix $C_{T}A^{i},\;i\in \{1,\ldots ,n-1\}$. Let $MR=\{C_{T}A^{i_{1}},C_{T}A^{i_{2}},C_{T}A^{i_{k}}\}$ be the set of the matrices with minimum rank.
11. Let $X_{j}=\{b_{j1},b_{j2},\ldots ,b_{jr}\}$ be the set of vertices correspond to the set of of maximum independent columns in $C_{T}A^{i_{j}}\in MR$.
12. Let $Y=\cup_{j\in \{1,2,\ldots ,k\}}X_{j}$. For each y∈Y, let R(y) denote the rank of the control matrix $CM(A,B_{\left\{y\right\}},C_{T})$ and define *R_max_* as follows:
  $$R_{\max}=\mathrm{maxR}(y),\;y\in Y.$$
  Considered the vertex y∈Y such that $R(y)=R_{\max}$. If more than one *y* exists with this condition, select *y* which has the highest ordering between the vertices of the graph *G*.
13. Let *S* be the all targets in *T* which are controlled by set {*y*} that are obtained by finding the maximum independent rows of the control matrix $CM(A,B_{\left\{y\right\}},C_{T})$.
14.
T=T−S

15. Remove the row and column corresponding to the vertex *y* from *A* and update *A*.
16.
D=D∪{y}

17. Go to Step 8.

*Part two of the algorithm:*

Let *T* be the original target set. Despite that each vertex in *T* is control by at least one vertex in D, it is possible that the Kalman’s rank-controllability condition may not be satisfied for the control matrix $CM(A,B_{D},C_{T})$. To tackle this issue, we do the following steps:

1. Let set *W* be the set of controlled target vertices corresponding to the maximum set of independent rows of the control matrix $CM(A,B_{D},C_{T})$. Let TC=T−W.
2. If TC=∅, go to the part three of algorithm. Otherwise:
3. For each $p_{i}\in TC,$ compute the rank of control matrix $CM(A,B_{D_{p_{i}}},C_{T})$ such that: $D_{p_{i}}=D\cup \{p_{i}\}$. Let $R(p_{i})=\mathrm{rank}(CM(A,B_{D_{p_{i}}},C_{T}))$. Let *p*_0_ be a vertex in *TC* such that $R(p_{0})$ be the maximum among all $R(p_{i}),\;p_{i}\in TC$.
4.
$D=D\cup \{p_{0}\}$ .
5. Go to the step 1.

*Part three of the algorithm:*

To obtained the minimal set of driver vertices we removed some of the vertices from D such that by removing them the Kalman condition is still valid. Let $D=\{d_{0},d_{1},\ldots ,d_{p-1}\}$.

1. For i=0,1,…,p−1
2.
$D_{\mathrm{temp}}=D-\{d_{i}\}$

3. If matrix $CM(A,B_{D_{\mathrm{temp}}},C_{T}))$ is full rank, $D=D-\{d_{i}\}$. Go to step 1
4. Return the set *D*.

To demonstrate the process of identifying driver vertices using the proposed GTCA algorithm, We analyzed a network *G*(*V*, *E*) with 8 vertices {1,2,3,4,5,6,7,8} and 7 edges E={(1,2),(4,3),(4,5),(6,3),(6,5),(7,4),(8,6)}, as shown in Supplementary Fig. S.1. The target set T={1,2,3,4,5,6} was selected for control. In this example, the set {1,3,7,8} satisfies both Kalman’s controllability rank condition and the exact controllability requirements for the target set. In contrast, the set {1, 7, 8} can structurally control the target set but does not meet Kalman’s rank condition. Thus, to achieve exact controllability, four driver vertices are required.

### 2.2 Time complexity of GTCA

The computational complexity of our method is analyzed in detail in the supplementary file. The overall time complexity of the proposed algorithm is determined to be $\max\{O(M^{2}N^{3}),O(M^{3}pN),O(M^{2}p^{2}N)\}$. Also to illustrate the algorithm’s computational performance, Supplementary Table S1 presents the execution times of the GTCA algorithm on well-known and widely used real-world networks.

## 3 Results

To evaluate the performance of the proposed GTCA algorithm, we compared it against three existing target controllability methods: BAGA, GeneticAlg, and TCMM. We applied these methods to various networks across different domains. To ensure a fair comparison, we focus on structural networks where both exact and structural control approaches can be applied, allowing us to clearly contrast the results and methodologies. We will address this by concentrating on cases where all edges have a weight of one. The networks analyzed fall into three categories:

### 3.1 Real networks

This category includes well-known and widely used networks from various sectors, such as: Social Networks (e.g. Prison network (Van Duijn *et al*. 2003)), Neuronal Networks (C.Elegans (Watts and Strogatz 1998)), Food Webs (Silwood (Montoya and Sol 2002), Mangrove (Ulanowicz and DeAngelis 1999)), Electronic Circuits (S208 (Milo *et al*. 2002), S420 (Milo *et al*. 2002)), Transcription Networks (Ecoli (Shen-Orr *et al*. 2002)).

### 3.2 Breast cancer-related networks

These networks are extracted from aggregated signaling pathways related to breast cancer from the KEGG database, in two different sizes.

### 3.3 Directed PPI networks

This category includes networks corresponding to three types of cancer: Breast Cancer (DEF, HCC-1428, and MDA-MB-361), Ovarian Cancer (DEF, OVCA8, and O19468), Pancreatic Cancer (DEF, Kp-3, AsPC) (Popescu *et al*. 2022).

In the first category, 50% of the network’s vertices were considered control targets, selected using two methods: Random target selection and local target selection. The results were calculated 20 times independently. In breast cancer signaling pathways, four sets of genes with different p-values were considered control targets. In directed PPIs, the set of essential proteins was considered the control targets. The results for the four algorithms are shown in Table 1. For each network, the table lists its type, number of nodes (|V|), number of edges (|E|), average degree (<k>), size of the target set (|T|), and the method of target selection. The ratio of drivers to target nodes is presented for each algorithm, with the best result highlighted in bold. Also, the results are illustrated in boxplots in Supplementary Figs S2–S5. Across all networks, the GTCA algorithm consistently demonstrates a lower ratio of driver nodes to target nodes compared to TCMM, despite both methods accounting for Kalman’s controllability condition. This shows that GTCA achieves target controllability with fewer driver nodes while maintaining robustness against network dynamics. While BAGA and GeneticAlg focus solely on structural controllability without considering the Kalman condition, GTCA often performs better or comparably. Notably, GTCA consistently outperforms GeneticAlg across almost all network types, showing a lower ratio of driver nodes to target nodes. When comparing GTCA to BAGA, the performance varies depending on the network type. In cancer PPI networks, GTCA outperforms BAGA by achieving a lower driver-to-target ratio, whereas in the Erdos-Renyi network, BAGA performs better with a more favorable ratio than GTCA. In other networks, the results are mixed, with GTCA occasionally outperforming BAGA, although the differences are generally small. Overall, GTCA shows greater robustness in handling complex network dynamics, particularly in scenarios where exact controllability is crucial.

**Table 1.** The ratio of driver nodes to target nodes is presented for each algorithm, with the best result highlighted in bold.

Network features						Target details		Algorithm			
		Name	—V—	—E—	<k>	—T—	Targets_selection	BaGA	GeneticAlg	TCMM	GTCA
							Random	**0.10**	0.21	0.28	**0.10**
Real_network	S.N	prison	67	182	5.43	33	Local	**0.04**	0.20	0.34	0.13
							Random	**0.17**	0.23	0.31	0.19
	N.N	C.Elegans	306	2345	15.33	153	Local	**0.12**	0.33	0.29	0.21
							Random	0.81	**0.78**	0.82	0.82
		Silwood	154	370	4.81	77	Local	0.8	**0.79**	0.82	0.81
							Random	0.24	0.18	0.60	**0.14**
	F.W	Mangrove	97	1492	30.76	48	Local	**0.15**	0.22	0.72	0.21
							Random	**0.22**	0.30	0.30	0.27
		S208	122	189	3.10	61	Local	**0.15**	0.30	0.24	0.24
							Random	**0.19**	0.39	0.29	0.30
	E.C	S420	252	399	3.17	126	Local	**0.15**	0.27	0.28	0.22
							Random	**0.67**	0.68	0.81	0.72
	Tr	EColi	423	578	2.73	211	Local	**0.62**	0.65	0.71	0.80
BC_Gene		Breast_956	956	6213	6.4	58	0.05	0.03	**0.02**	0.03	**0.02**
						157	0.01	**0.15**	0.19	0.19	0.18
						119	0.005	**0.11**	0.14	0.13	0.13
		Breast_478	478	2055	4.2	50	0.001	0.04	**0.03**	0.04	**0.03**
		DEF	1415	2435	3.44	112		0.68	**0.53**	0.68	0.63
Cancer_PPI		HCC-1428	1495	2650	3.54	126		0.67	0.51	0.66	**0.48**
	Breast	MDA-MB-361	1478	2590	3.50	124		0.69	0.51	0.67	**0.49**
		DEF	1047	1579	3.01	140		0.75	0.66	0.75	**0.63**
		OVCA8	1157	1781	3.07	161	Essential proteins	0.73	0.63	0.72	**0.60**
	Ovarian	O19468	1047	1579	3.01	159		0.70	0.66	0.74	**0.62**
		DEF	991	1484	2.99	168		0.74	0.67	0.69	**0.62**
		KP-3	1134	1757	3.09	167		0.74	0.64	0.70	**0.62**
	Pancreatic	AsPC	1022	1534	3.0	125		0.75	0.63	0.67	**0.60**

Network topology also affects the efficacy of the control algorithm. The execution time of GTCA tends to be longer in dense networks compared to sparse ones. Additionally, Table 1 shows that the ratio of the number of drivers to the number of targets increases as the network’s density decreases. This means that controlling sparse networks requires more driver vertices compared to dense networks. For example, in the dense Mangrove network, the ratio of drivers is much lower than in the sparse Silwood network. Furthermore, networks with a larger scale-free coefficient also require more driver vertices, in contrast to networks that fit the Erdos-Renyi model. In the scale-free Ecoli network, the ratio of drivers is significantly higher than in the Erdos-Renyi S420 network. To better analyze the influence of network topology, we compared the average degree, average closeness, average betweenness, and average eigenvector centrality of different networks with the corresponding averages for the driver vertices identified by GTCA (Supplementary Table S3). This table shows that, in general, these metrics are quite similar, although the averages for the set of driver vertices are usually slightly lower compared to the averages for the whole network. For a more comprehensive understanding of how the algorithms perform under varying conditions, we also evaluated the four algorithms by considering target sets comprising 5% and 10% of the vertices in the analyzed real networks, as presented in Supplementary Table S2. In these scenarios, the results obtained by the GTCA algorithm were even better than those of the other methods, particularly when compared to the 50% target vertex scenario. These findings demonstrate the superior performance and adaptability of GTCA across different conditions and network types.

Although it is generally not possible to compare the results of structural and exact control approaches, in structural networks, the number of driver vertices required for exact controllability is at least equal to, and often greater than, that required for structural controllability. On the other hand, integrating a method that achieves exact controllability with one that achieves structural controllability has the potential to outperform both methods individually. With this in mind, we combined the GTCA and BAGA approaches to enhance their overall performance. In this combined framework, the driver vertices identified by the GTCA method serve as the control target vertices for the BAGA method. The results of this two-step target control process, applied to real-world networks, are compared with the outcomes of the original BAGA method in Table 2. These results confirm a substantial improvement compared to using BAGA alone. Notably, the set of driver vertices identified by GTCA ensures exact controllability, and when utilized as the target control in BAGA, the driver vertices identified by BAGA ensure structural controllability. Table 2 showcases the outcomes of applying this scenario to seven real-world networks. The columns NBAGA and N2stepCtrl represent the ratio of the number of obtained driver vertices to the network size for BAGA alone and the combination of GTCA and BAGA methods, respectively, under both random and local target selection protocols. In most cases, the integrated GTCA-BAGA algorithm exhibited markedly improved results compared to using BAGA alone. Thus, this two-step method can be deemed a viable solution for acquiring fewer driver vertices in the target control problem.

**Table 2.** Results comparing the implementation of the combined GTCA and BAGA methods (N2stepCtrl column) with BAGA alone (NBAGA column) for controlling 50% of nodes in real networks under both random and local target selection protocols.

Network	Random selection		Local selection	
name	N_*BAGA*_	N_2__*stepCtrl*_	N_*BAGA*_	N_2__*stepCtrl*_
Prison	0.06	0.02	0.03	0.03
c-elegans	0.09	0.07	0.03	0.03
E.Coli	0.33	0.26	0.31	0.21
mangrove	0.12	0.03	0.10	0.02
Silwood	0.41	0.39	0.39	0.39
S208a	0.13	0.04	0.09	0.02
S420a	0.14	0.04	0.11	0.03

The table presents the ratio of driver nodes to target nodes for each network.

## 4 Case study

In this section, we focus on the biological analysis of driver proteins identified using the GTCA method. We examined three directed PPI networks associated with breast cancer: Breast-DEF, Breast-HCC1428, and Breast-MDA-MB-361. The identification of control targets within these networks was facilitated by referencing the COLT-Cancer database, which provides essential genes across various human cancer cell lines (Koh *et al*. 2012). These networks contain 1415, 1495, and 1478 vertices, respectively, with 112, 126, and 124 target vertices identified within them.

Given the stochastic nature of the GTCA algorithm, we ran it ten times on each network. To ensure the reliability of the results, we considered only those driver proteins that appeared in at least nine out of ten iterations. The proteins that consistently emerged as drivers in these runs are listed in Supplementary Table S4. Across the repeated GTCA runs, 49, 46, and 46 proteins were identified as drivers for controlling essential genes in the Breast-DEF, Breast-HCC1428, and Breast-MDA-MB-361 networks, respectively. By identifying common proteins across all three networks, we narrowed our focus to 68 unique proteins for further analysis.

To further understand the role of these driver proteins and their network connectivity, we conducted a hub centrality analysis. We constructed a PPI network comprising the 68 unique proteins using data from the STRING database, considering only physical and functional connections with a confidence score of at least 0.7. The resulting network, shown in Supplementary Fig. S6, exhibits a modular structure, with several isolated proteins and a cohesive largest connected component due to the stringent edge selection criteria. Within this network, several driver proteins emerged as central hubs, demonstrating high connectivity. Notably, the NCBP1 protein exhibited the highest degree with nine connections, followed by HSP90AA1 with seven connections, RPL5 with six, and both RPS3 and RPL11 with five connections each. These findings highlight the central roles these proteins play in mediating interactions within the network. Following the identification of these central hub proteins, we examined their biological significance. These hub proteins, characterized by their high connectivity, are critical to various cellular processes and may serve as potential therapeutic targets in cancer treatment. To explore potential therapeutic interventions targeting these hub proteins, we consulted the Drug–Gene Interaction Database. We identified the most relevant drug for each hub protein and elucidated its mechanism of action using data from the MedChemExpress database (see Supplementary Table S5).

This analysis identified four drugs, three of which—Doxorubicin hydrochloride, Dorlimomab aritox, and Exaluren—are already established in cancer treatment. These drugs target pathways associated with the identified hub proteins, offering potential therapeutic interventions in cancer management. Additionally, Obefazimod, which targets the NCBP1 protein, presents a promising candidate for drug repurposing in cancer therapy, offering new therapeutic possibilities beyond its original indication. This network-based approach to drug selection provides valuable insights into potential therapeutic strategies for cancer and underscores the significance of pharmacological interventions that target key proteins in disease management.

## 5 Discussion

In recent years, network theory has become an indispensable tool for modeling complex systems across various scientific disciplines, especially in the biological sciences. Complex networks represent system variables as vertices and their interactions as edges, offering a powerful framework for analyzing intricate systems. This approach, grounded in graph theory, enables deep exploration of network structures, allowing for detailed comparisons across states, sub-network analyses, module identification, and the pinpointing of critical vertices and edges. Unlike traditional methods, network-based approaches effectively capture both linear and non-linear relationships, providing richer insights into the dynamics and interactions within complex systems. The integration of control theory with network analysis has expanded the applications of network science, particularly in managing systems with many variables. Network controllability focuses on identifying key variables that can influence target variables, enabling the steering of the system towards desired states. Initially applied in bioinformatics for drug target identification, network controllability is now used in engineering, social sciences, and economics. In cases where controlling all variables is impractical, identifying driver vertices that can control target vertices with minimal energy is critical for efficient control strategies in complex systems. In practical cases, full control of all system variables is often unnecessary, giving rise to the target control problem, where only a subset of variables (target vertices) needs to be controlled. The GTCA algorithm was designed to address this challenge by providing precise control in complex networks, leveraging Kalman’s controllability rank condition to ensure exact control. Structural controllability is based on the network’s topology and considers only the presence or absence of edges. While structural controllability offers a qualitative solution by assessing connectivity, it does not guarantee precise control of the network’s dynamics. In contrast, Kalman’s Rank Condition provides a more rigorous framework by incorporating both structural and dynamic properties of the network. For example, while structural controllability might suggest that a complete network can be controlled with one driver vertex, Kalman’s condition may require signals to be applied to all vertices except one of them, for exact control. In this study, we used unweighted networks to align with previous methods, ensuring consistency for comparison. In such cases, assuming no changes in edge weights, driver vertices identified through exact controllability can control the network to any desired point in the change space, encompassing all vertices. While driver vertices identified through structural control can manage nearly all points in the state space, there may still be certain points that cannot be reached.

On the other hand, by using the driver vertices identified by the GTCA algorithm as inputs for structural controllability algorithms like BAGA, we can further refine the control strategy. This hybrid approach not only minimizes the impact on non-target vertices but also optimizes the overall control process, resulting in shorter control paths, fewer steps, and reduced costs. Combining GTCA with structural control methods creates a synergistic effect, significantly reducing the number of driver vertices needed for effective control. This integrated approach enhances both the robustness and scalability of control strategies across diverse network configurations, making it especially valuable in areas like network biology and communication systems, where precision and efficiency are essential. Building on this foundation, we extended the GTCA algorithm’s application to drug repurposing, a crucial area in modern medicine that offers cost-effective alternatives to developing new drugs. Network-based analysis, driven by the GTCA algorithm, facilitates the identification of driver proteins that can influence disease-related proteins toward a healthier state. In a breast cancer case study, we applied the GTCA algorithm to identify driver proteins within protein-protein interaction (PPI) networks. These driver proteins were then targeted to modulate essential proteins within the network. Using this network-based approach, we constructed a secondary PPI network linking driver proteins with highly connected proteins, effectively mapping potential drug targets. This allowed us to identify drugs that target proteins connected to the driver proteins, highlighting their potential as novel therapeutic agents. These findings emphasize the significant potential of integrating network control strategies with drug discovery, offering new paths for treating complex diseases like breast cancer. Throughout this study, we demonstrated the robust capabilities of the GTCA algorithm in achieving precise control over target vertices within complex networks. Our comparative analysis shows that GTCA consistently outperforms existing algorithms, requiring fewer driver vertices and thereby improving control efficiency. Different controllability algorithms, including both structural and exact controllability approaches, tend to perform better in dense networks compared to sparse ones, and generally show better results in random networks than in scale-free networks. Our algorithm follows this trend but stands out in sparse networks with a high scale-free coefficient. This advantage arises because structural controllability algorithms rely on maximum matching and greedy selection, focusing solely on the network’s structural properties without considering dynamic behavior. In sparse and scale-free networks, where connectivity varies significantly, structural controllability often selects high-degree nodes, leading to suboptimal control configurations. In contrast, the GTCA algorithm incorporates Kalman’s exact controllability condition, ensuring that driver vertices are selected based on both structural and dynamic properties, which is particularly important in networks with heterogeneous node distributions, such as biological systems. Despite the strengths of our approach, it is important to acknowledge that target control in complex networks remains an NP-hard problem, requiring efficient solution strategies. Future research should focus on enhancing this approach by developing meta-heuristic methods and identifying smaller, manageable network subsets capable of effectively controlling desired targets, ensuring the continued evolution of network control strategies across various domains.

## 6 Conclusion

In many dynamical systems, it is often impractical to apply control signals to every component, making it crucial to identify the smallest set of vertices capable of exerting hierarchical control based on the network’s topology. While exact controllability, as defined by Kalman’s controllability rank condition, poses significant challenges in large networks, solutions from structural controllability frequently fail to meet these demands. As a result, no existing method effectively pinpoints the necessary driver vertices for exact controllability across such complex networks. Target control—whether exact or structural—constitutes an NP-hard problem, presenting a significant challenge in network controllability. To address this, we introduced the GTCA algorithm, which leverages matrix rules and linear algebra to efficiently identify the minimal set of driver vertices necessary for control. By meeting Kalman’s controllability rank condition, GTCA offers a robust solution that significantly enhances performance compared to existing algorithms, particularly in controlling desired targets with fewer driver vertices. For target controllability, no deterministic polynomial-time solution exists. All methods discussed in this paper are heuristic in nature, aiming to find optimal solutions without guaranteeing them. These methods typically reach a local optimum rather than a global one. Given that obtaining a global optimum for the driver set in large networks is infeasible, comparing results across different methods becomes essential. Our comparisons indicate that GTCA produces promising results even if it achieves only a local optimum. In many cases, this local optimum is superior to those obtained by other methods. Specifically, GTCA leverages matrix analysis and linear algebra to achieve promising results across networks of varying sizes, including large networks. Additionally, when comparing GTCA to the BAGA method, which structurally controls networks, the effectiveness of GTCA is evident. The application of GTCA in identifying key driver proteins in biological networks, especially in the context of breast cancer, underscores its potential for real-world applications. By applying network-based analysis and leveraging controllability principles, GTCA not only identifies critical nodes within these networks but also facilitates the exploration of therapeutic interventions, including drug repurposing, for disease management. Looking ahead, further advancements in network controllability algorithms like GTCA hold great promise for tackling complex control challenges in large networks. By continuing to integrate innovative approaches with established principles, we can deepen our understanding and improve our ability to manipulate complex systems across a wide range of scientific disciplines, from biology to engineering and beyond.

## Supplementary Material

btae630_Supplementary_Data

## Data Availability

The data underlying this article are available in the article and in its online supplementary material.
