# Supplementary material for: Target controllability: a feed-forward greedy algorithm in complex networks, meeting Kalman’s rank condition
Source: Bioinformatics. 2024 Oct 23;40(11):btae630. doi: 10.1093/bioinformatics/btae630 (PMC11568069; doi:10.1093/bioinformatics/btae630)
Supplement: btae630_Supplementary_Data [file btae630_supplementary_data.pdf]

Supplementary

Target Controllability: A Feed-Forward Greedy Algorithm in Complex Networks,

Meeting Kalman’s Rank Condition

Example

Figure $S.1$ illustrates the process of identifying driver vertices using the proposed GTCA algorithm. We analyzed a network $G(V, E)$ with *8* vertices $\{1,2,3,4,5,6,7,8\}$ and *7* edges $E = \{(1,2),(4,3),(4,5),(6,3),(6,5),(7,4),(8,6)\}$, as shown in Figure $S.1-(a)$. In this network, the vertices $T = \{1,2,3,4,5,6\}$ were selected as the target vertices for control, indicated in green.

In the first step, the algorithm identifies vertex *1*, a target vertex with an in-degree of zero, and adds it to the driver set *D*. At this stage, as shown in Figure $S.1-(b)$, the driver set $D = \{1\}$ is capable of controlling vertices $\{1,2\}$. Next, the algorithm identifies the driver vertex that can control the maximal number of remaining target vertices. Vertex *7* is selected, and the driver set is updated to $D = \{1,7\}$. This new driver node can control vertices $\{4,5\}$, as shown in Figure $S.1-(c)$. In the following step, the algorithm adds vertex *8* to the driver set *D*, as depicted in Figure $S.1-(d)$. This vertex can control the remaining target vertices $\{3,6\}$. After these steps, the algorithm calculates the rank of the control matrix $CM(A, B\_D, C\_T)$. However, this system does not satisfy Kalman's rank-controllability condition.

To address this, in the second module of the algorithm, vertex *3* is added to the driver set, resulting in $D = \{1,3,7,8\}$. Now, the matrix $CM(A, B\_D, C\_T)$ is full rank, as shown in Figure $S.1-(e)$.

In the third module, the algorithm checks if any driver in D can be removed while still satisfying Kalman's rank-controllability condition. In this example, none of the driver nodes in D can be removed without violating the condition. Consequently, the final driver set output by the algorithm is $D = \{1,3,7,8\}$.

In the second module of the algorithm, we verify Kalman's rank-controllability condition. If the initially identified set of candidate driver nodes does not satisfy this condition, we augment the set by including additional target nodes until Kalman's condition is met. In the third module, we derive a minimal set of driver nodes that satisfy Kalman's condition by iteratively removing nodes from the candidate set obtained in the second module, ensuring Kalman's condition remains satisfied.

In the provided example, the set $\{1,3,7,8\}$ satisfies both Kalman's controllability rank condition and the requirements for exact controllability. On the other hand, while the set $\{1,7,8\}$ can structurally control the network, it does not meet Kalman's controllability rank condition. To meet the Kalman condition and achieve exact controllability, the network requires four driver vertices.

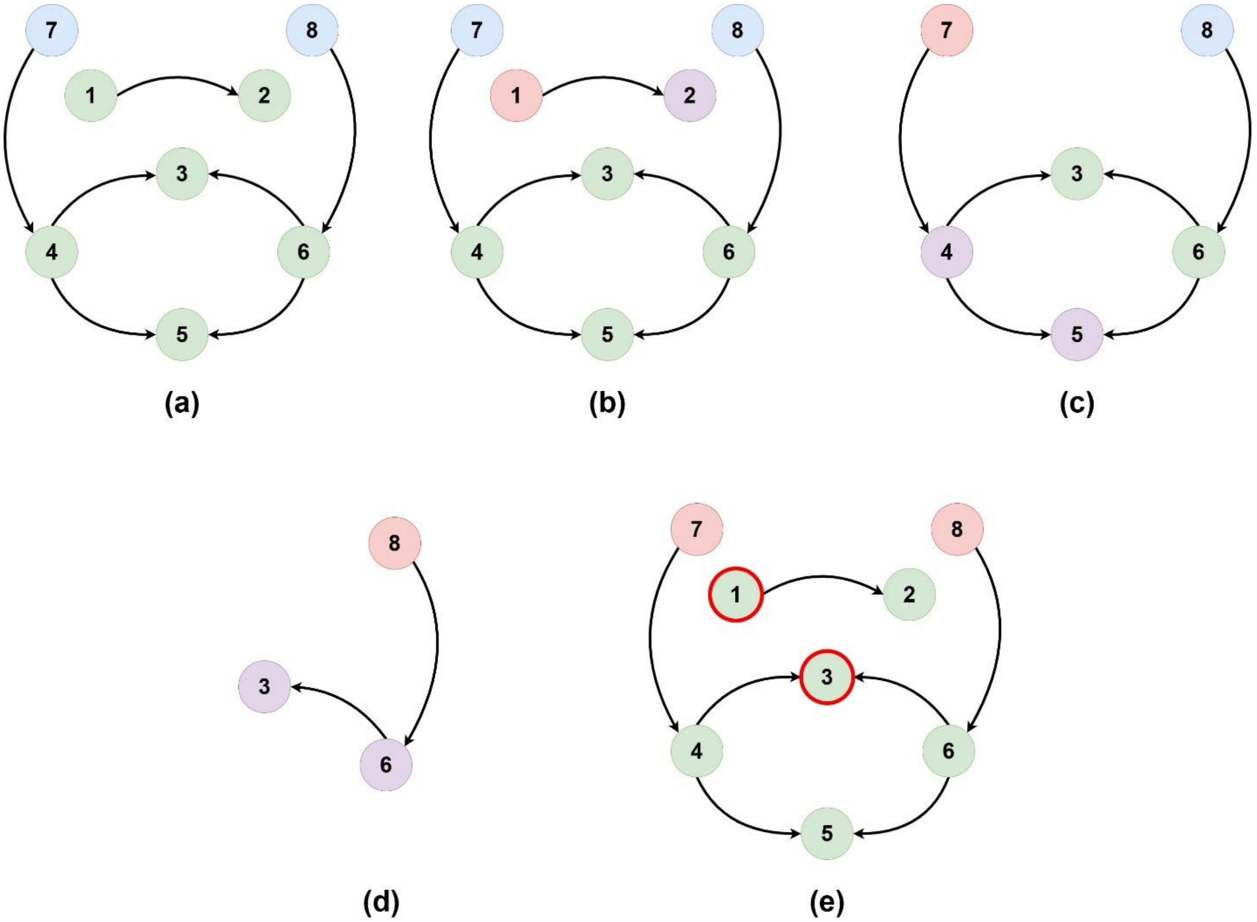

Figure S.1: Identification of driver nodes by the GTCA algorithm. (a) A directed network with eight nodes and target set $T = \{1,2,3,4,5,6\}$, indicated in green. (b) Node *1* is added to the driver set, marked in red, and vertex *2*, controlled by it, is colored purple. (c) Nodes $\{1,2\}$ are removed. Node *7*, which has the highest control power for the remaining vertices, is added to the driver set, and nodes $\{4,5\}$ are under its control. (d) Nodes $\{7,4,5\}$ are removed, and node 8 is added to control the remaining target nodes. (e) Although each target node is under the control of at least one driver node, the set $\{1,7,8\}$ does not satisfy Kalman's rank-controllability condition. Therefore, node *3* is added to the driver set. By removing any node from the driver set $\{1,3,7,8\}$, Kalman's rank-controllability condition would no longer be satisfied, making this the minimal driver set.

Analysis of Time Complexity for the Proposed Algorithm

In this section, we provide a detailed analysis of the time complexity of our novel algorithm. The algorithm consists of several key steps, each contributing to the overall computational complexity. We analyze each step individually and then combine the results to determine the total time complexity. In part 1, the algorithm identifies the driver nodes with the minimum number necessary to ensure that all target nodes are covered—meaning each target node is controlled by at least one driver node. Since the initial driver set may not satisfy Kalman's rank-controllability condition, part 2 adds nodes to the driver set to meet this condition. Finally, in part 3, the algorithm removes nodes from the driver set obtained in the previous step, ensuring that Kalman's rank-controllability condition remains satisfied while minimizing the size of the driver set.

Part 1

Step 1: Initializing an Empty Set

The operation of initializing an empty set is performed in constant time, denoted as $O(1)$.

Step 2: Finding Vertices with In-degree of Zero

The overall time complexity for finding nodes with an in-degree of zero in a directed graph is $O(N + E)$, where N is the number of vertices and E is the number of edges.

This complexity is optimal since each node and edge must be inspected at least once to determine the in-degrees and identify nodes with zero in-degree.

Step 3: Checking if a Set is Empty

This operation is performed in constant time, denoted as $O(1)$.

Step 4: Creating the Control Matrix (*CM*); $CM(A, B_D, C_T)$.

This step involves multiplying a square matrix A of size N×N by itself N times. Thus, the total time complexity for this step is $O(N^4)$.

Step 5: Finding the Maximum Set of Independent Rows of CM of Size $M \times pN$ where p denoted the number of drivers obtained until now.

The time complexity for this operation is $O(M^2pN)$. Given $p \leq M$, this simplifies to $O(M^3N)$.

Step 6: Deleting Elements Using a Linked List

This operation is of order $O(1)$.

Step 7: Deleting Rows and Columns

Deleting specific rows and columns of CM has a time complexity of at most $O(MN)$.

Step 8: Checking if a Set is Empty

Performed in constant time, $O(1)$.

Step 9: Construction of at Most *M* Matrices

The time complexity of constructing these matrices is $O(MN^3)$.

Step 10: Computing the Rank of Matrices of Size $M \times N$.

When $M \leq N$, the time complexity is $O(M^2N)$.

Step 11: Finding the Maximum Set of Independent Columns

The time complexity for finding the maximum set of independent columns in *N* matrices is $O(N^4)$.

Steps 12, 13, 14, 15, and 16

These steps have a time complexity of $O(1)$ each.

Since Steps 8 to 16 are repeated at most *M* times, the time complexity for this part of the method is at most $M(MN^3 + M^2N)$. Given that $M \leq N$, the overall time complexity of the first module of the method is $M^2N^3 + MN^3 = O(M^2N^3)$.

Part 2

Step 1: Finding the Maximum Set of Independent Rows of *CM* of Size $M \times pN$

The time complexity is $O(M^2 \times pN)$.

Step 2: If *CM* is Full Rank

If *CM* is full rank, we proceed to Part 3 of the method. Otherwise, Step 1 is repeated at most M times, so the time complexity of Part 2 is $O(M^3 \times pN)$.

Part 3

Computing the Rank of a Matrix of Size $M \times pN$

This operation has a time complexity of $O(M^2 \times pN)$. Since this part is repeated at most p times, the overall time complexity of this part is $O(M^2 \times p^2N)$.

By combining the complexities from all parts, the overall time complexity of the proposed algorithm is max $\{O(M^2N^3), O(M^3 \times pN), O(M^2 \times p^2N)\}$. Since $p \leq M \leq N$ we can conclude that the algorithm has a worst-case time complexity of $O(N^5)$. However, in most practical scenarios where $p \leq M \ll N$, the time complexity for large networks with a small number of targets reduces to $O(N^3)$. This detailed analysis highlights the dominant terms contributing to the computational requirements, ensuring an accurate and comprehensive evaluation of the algorithm's efficiency. It is important to note that simply checking Kalman's controllability rank condition is of the order $O(N^3)$, and every exact controllability algorithm must perform this check. Therefore, while the GTCA algorithm may be slower than structural controllability algorithms, its worst-case complexity of $O(N^5)$ remains a suitable order for addressing the exact controllability problem.

In addition to analyzing computational complexity, we evaluated the control effort and control performance of the algorithm as functions of the time required for various complex networks. Control effort refers to the amount of input needed to drive the system to the desired state, while control performance measures the effectiveness in achieving the control objectives. Our analysis reveals that for dense networks, the control effort tends to be higher due to the increased number of connections that need to be managed. Conversely, sparse networks, while often requiring more driver nodes, exhibit lower control effort per node because fewer

connections need simultaneous management. However, the overall control effort in sparse networks can still be higher due to the increased number of driver nodes required. Control performance, measured by the speed and accuracy in reaching the target state, generally improves in networks with well-defined hierarchical structures and higher connectivity.

To further illustrate the algorithm's computational performance, Table S.1 presents the execution times of the GTCA algorithm on a selection of well-known and widely used real-world networks. The table lists the number of nodes, average degree, and total execution time (in seconds) for each network, providing insight into how GTCA performs across different network topologies and sizes. The results demonstrate that while GTCA is computationally intensive, it remains feasible for a wide range of network types, from those with a small number of nodes to those with complex and dense structures. In summary, although the GTCA algorithm exhibits higher time complexity in its worst-case scenario, its effectiveness in ensuring exact controllability, particularly for large and complex networks, justifies the computational cost.

Table S.1: Execution times of the GTCA algorithm on a selection of well-known and widely used real-world networks. The table lists the number of nodes, average degree, and total execution time (in seconds) for each network. These results provide insight into the algorithm's computational performance across different network topologies and sizes.

Networks	Number_of_Nodes	Avg_Degree	Total time(s)
Prison	67	4.23880597	**4.2211**
S208	122	3.098360656	**14.0165**
Mangrove	97	29.81443299	**26.6921**
Silwood	154	4.805194805	**52.3867**
S420	252	3.166666667	**536.4475**
E.Coli	423	2.73286052	**3319.2663**
C.Elegans	306	14.03921569	**4630.4397**

Boxplots of Results

Figures *S.2* and *S.3* present box plots corresponding to the results of executing the four algorithms on the *C-Elegans network* and the pancreatic *Kp-3 PPI network*, respectively, using both local and random selection methods for the target selection. Additional box plots for other analyzed networks can be found in the Figures *S.4* and *S.5*.

Figure *S.2* shows that the GTCA algorithm outperforms the other methods in both random and local selection of target vertices, consistently achieving a lower number of driver vertices. While all algorithms perform better with local target selection compared to random selection, GeneticAlg exhibits the greatest variability in results across these selection strategies.

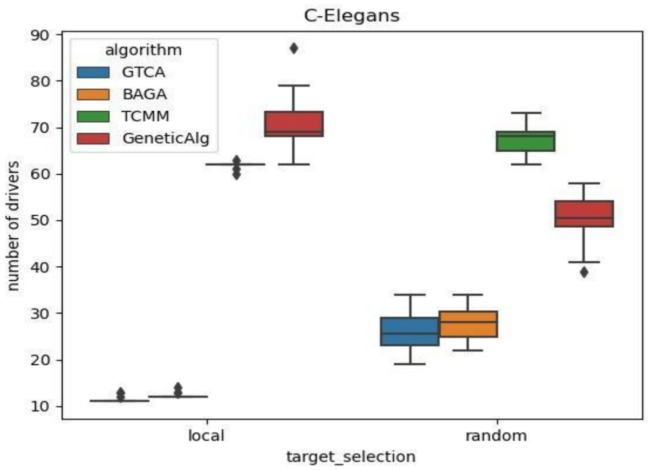

Figure S.2: Boxplot showing the number of driver nodes obtained by the GTCA (blue), BAGA (orange), TCMM (green), and GeneticAlg (red) algorithms on the *C-Elegans network*. Each algorithm was executed *20* times independently to control the target nodes specified in Table 1. The results indicate that the GTCA consistently identifies fewer driver nodes compared to the other algorithms.

Figure *S.3* compares the four algorithms on the pancreatic *Kp-3 PPI network*, further underscoring the superiority of GTCA. The results indicate that the GTCA algorithm is capable of controlling a target set of *167* vertices in this network, which totals *1134* vertices, using approximately *100* driver vertices on average.

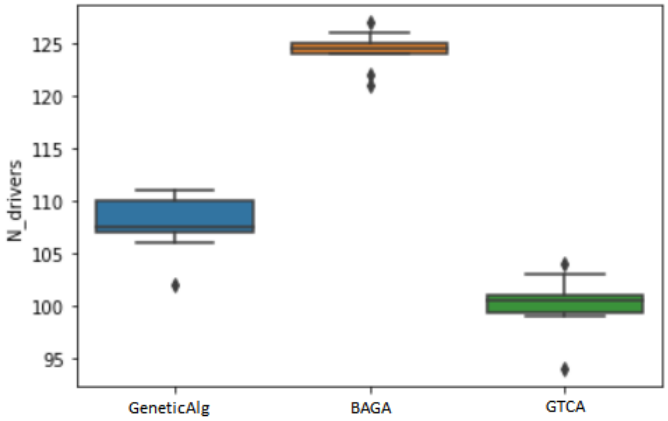

Figure S.3: Boxplot showing the number of driver nodes obtained by the GeneticAlg (blue), BAGA (orange), and GTCA (green) algorithms on the pancreatic *Kp-3 PPI network*. Each algorithm was executed *20* times independently to control the target nodes specified in Table 1. In this network, the GTCA consistently identifies a more appropriate number of driver nodes compared to the other algorithms.

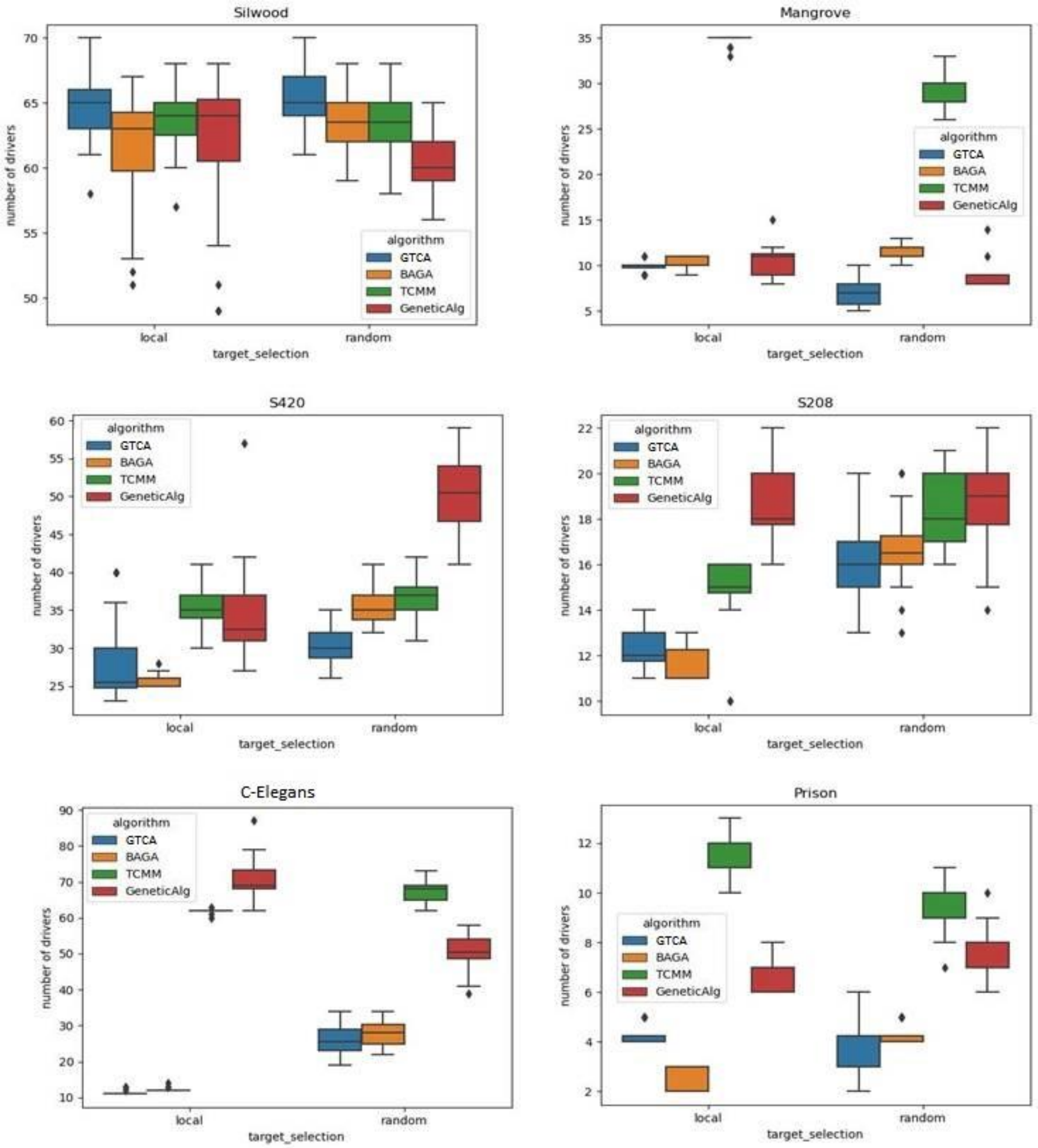

Figure S.4: Boxplots showing the performance comparison of four different algorithms—GTCA, BAGA, TCMM, and GeneticAlg—across seven real-world networks. The results illustrate the effectiveness of each algorithm in identifying optimal driver nodes for network control, highlighting the variance in performance across different network topologies.

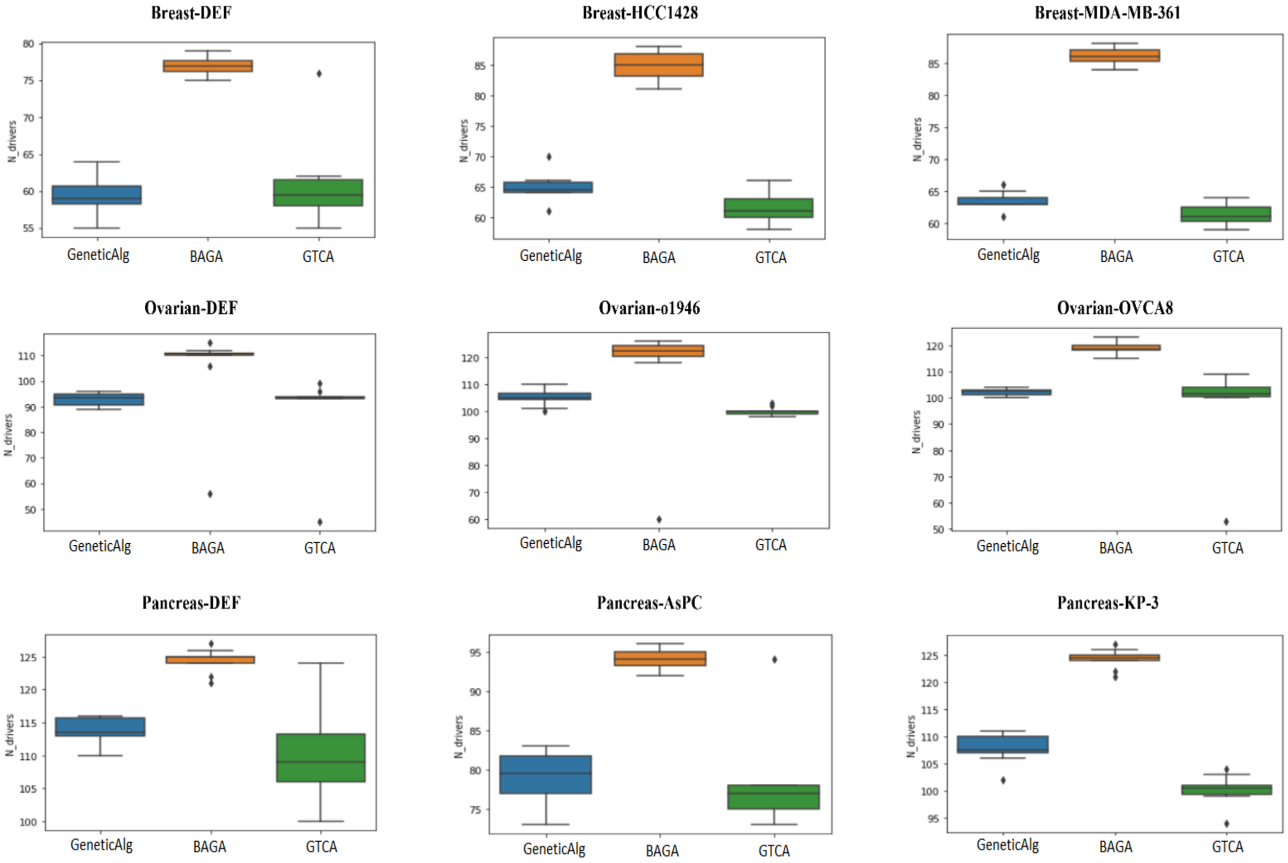

Figure S.5: Boxplots depicting the performance comparison of three different algorithms—GeneticAlg, BAGA, and GTCA—on protein-protein interaction (PPI) networks related to breast cancer, pancreatic cancer, and ovarian cancer. The plots demonstrate the relative efficiency of each algorithm in identifying driver nodes crucial for controlling these specific biological networks.

Table S.2: This table presents the results of implementing four target controllability algorithms—TCMM, BAGA, GeneticAlg, and GTCA—across various real networks, considering 5% and 10% of the network vertices as the control targets.

Target	**0.05**				**0.10**			
Algorithm<br>Network	TCMM	BAGA	GeneticAlg	GTCA	TCMM	BAGA	GeneticAlg	GTCA
Prison	0.0171	0.0171	0.0171	0.0171	0.0461	0.0970	0.0970	0.0368
Mangrove	0.0144	0.0134	0.0134	0.0134	0.0365	0.0853	0.0543	0.0298
Silwood	0.0454	0.0457	0.0457	0.0454	0.0855	0.0899	0.0855	0.0847
S208	0.0176	0.0171	0.0171	0.0176	0.0412	0.0934	0.0722	0.0315
S420	0.0121	0.0307	0.0321	0.0121	0.0198	0.0454	0.0275	0.0158
C.Elegans	0.0163	0.0478	0.0283	0.0163	0.0452	0.0957	0.0532	0.0426
E.Coli	0.0449	0.0477	0.0456	0.0410	0.0850	0.0912	0.0896	0.0803

Table S.3: Comparison of network features between the entire network and the identified driver nodes across various networks. The table presents average values for key metrics—degree, closeness, betweenness, and eigenvector centrality—showing both the overall network averages and the averages specific to the driver nodes selected by the GTCA algorithm. This comparison provides insights into the structural characteristics of driver nodes relative to the broader network topology.

Networks		Network_avg_features				Drivers_avarage_features			
Name	Selection	**Avg_ Degree**	**Avg_ Closeness**	**Avg_ Betweenness**	**Avg_ Eigenvector**	**driver_Avg_ Degree**	**driver_Avg_ Closeness**	**driver_Avg_ Betweenness**	**driver_Avg_ Eigenvector**
**Prison**	Random	4.23880597	0.304871787	0.036224472	0.096658822	3.538461538	0.290810992	0.029997222	0.064857181
	local					4	0.302218978	0.021334953	0.101398428
**Mangrove**	Random	29.81443299	0.597013411	0.007293362	0.091626413	31.47826087	0.610936754	0.014408857	0.0928353
	local					35.33333333	0.625116845	0.014126708	0.105098584
**S208**	Random	3.098360656	0.207611204	0.032731563	0.062872996	2.573770492	0.197300953	0.026328331	0.040101131
	local					3.076923077	0.207405197	0.032267039	0.05674638
**S420**	Random	3.166666667	0.175875383	0.019225574	0.039056956	2.595041322	0.166804421	0.012183802	0.021460839
	local					2.987804878	0.17691867	0.019508322	0.025944417
**C.Elegans**	Random	14.03921569	0.388333343	0.004509325	0.042317351	8.280701754	0.313866319	0.006315622	0.020642079
	local					17.78571429	0.385451175	0.024755982	0.032788597
**Silwood**	Random	4.805194805	0.298561369	0.015511149	0.042184081	3.742424242	0.289581839	0.004524069	0.038759803
	local					4.204379562	0.291977775	0.006531746	0.04162342
**E.coli**	Random	2.73286052	0.130645908	0.005476412	0.020153362	2.900302115	0.133320943	0.005496892	0.018520678
	local					2.854395604	0.134399426	0.005663422	0.020345592

**SignalingPathways**	size:956	12.73430962	0.2035322	0.004258536	0.009533199	2.854395604	0.134399426	0.005663422	0.020345592
**CancerNetwork (size:478)**	pval:001	8.585774059	0.141305406	0.007530117	0.015200027	2.854395604	0.134399426	0.005663422	0.020345592
	pval:0001					8	0.169045383	0.01776777	0.005049
	pval0005					6.753246753	0.163270794	0.011088189	0.002692184
**BreastCancer**	DEF	3.361130742	0.186811789	0.001801627	0.012767561	2.016949153	0.161601045	0.000530405	0.00711107
	HCC1428	3.459531773	0.191598522	0.00172374	0.012386537	2.365079365	0.164898844	0.000815539	0.007790756
	MDA-MB	3.422192152	0.183738862	0.001646651	0.012290282	2.253968254	0.154803317	0.000609211	0.007189798
**Ovarian**	DEF	2.93982808	0.176824106	0.002231731	0.01439903	2.29787234	0.113560903	0.001223805	0.004954474
	O1946	3.085714286	0.186605858	0.002151051	0.013774897	4.04	0.181240144	0.002371151	0.012130038
	OVCA8	3.00605013	0.185006478	0.002072343	0.01374121	2.131313131	0.119927311	0.001025683	0.004178508
**Pancreatic**	AsPC-1	2.945205479	0.167248977	0.002456305	0.013487365	2.906666667	0.126401393	0.002672551	0.006937679
	DEF	2.946518668	0.166698081	0.002452869	0.014608739	2.472222222	0.123667233	0.001506049	0.006861517
	KP-3	3.028218695	0.17867879	0.002345689	0.014099395	2.515463918	0.131941229	0.00176889	0.007812215

Additional Information on the Case Study

Table S.4: Identification of driver proteins in breast cancer protein-protein interaction (PPI) networks. The table lists proteins that were identified as driver nodes across multiple replicates, with columns indicating the frequency of each protein being selected as a driver (*9* times out of *10* replicates and *10* times out of *10* replicates) in different breast cancer subtypes (Breast-DEF, Breast-HCC1428, and Breast-MDA-MB-361). These results highlight key proteins potentially critical for controlling breast cancer-related PPI networks.

network	Proteins that were driver 9 times in 10 replicates	Proteins that were driver 10 times in 10 replicates
Breast-DEF	EFNA5, CDK11A, SRSF7, FBXW11, FIP1L1, EFNB1, PTPRZ1, XPO1, PSMA6, RPL5, KEAP1, BRD4, EIF3I, CDK12, PPP2R1A, DDX3X, SUPT5H, NUP98, PTPN22, MYBL2, MYH9, PRPF3, TUBA1C, VRK2, NR1D1	TNK2, GPC4, WNT11, COL1A1, NEK1, ANGPT1, MAPKAPK2, NUMB, PPP5C, MTCP1, ID1, NCBP1, TAOK3, NRG1, DDR2, KPNB1, SMG1, PLRG1, EIF2AK2, TUBB, RPL11, SF3B1, UBA1, NACA
Breast-HCC1428	GPC4, RLN2, MAPKAPK2, FBXW11, FIP1L1, PTN, DDB1, RPL5, XPO1, NRTN, EIF3I, PSMA6, PTPN22, EIF2B4, NR1D1, KEAP1, DYNLRB1	PPP2R1A, GABPB2, IL3, ANGPT1, SRSF7, NEK1, TESK2, COL1A1, EFNB1, NPPA, NCBP1, CDK12, TAOK3, ID1, IL23A, PLRG1, MTCP1, SMG1, NUMB, NRG1, BRD4, RPL11, TUBB, NUP98, PRPF3, NACA, RPS3, UBA1, TUBA1C
Breast-MDA-MB-361	GABPB1, GPC4, PLRG1, FBXW11, MAPKAPK2, COL2A1, SRSF3, NPPA, HSP90AA1, KPNB1, RPL5, SUPT5H, DDR2, EIF2B4, NR1D1, EFNB1, BRD4	FGF6, WNT11, TESK2, FIP1L1, PIM1, VRK2, ANGPT1, NEK1, COL1A1, NCBP1, EIF2AK2, NUMB, ADCYAP1, MTCP1, NRTN, NRG1, CDK12, RPL11, PPP2R1A, XPO1, TAOK3, ID1, SMG1, TUBB, NACA, PTPN22, TUBA1C, UBA1, NUP98

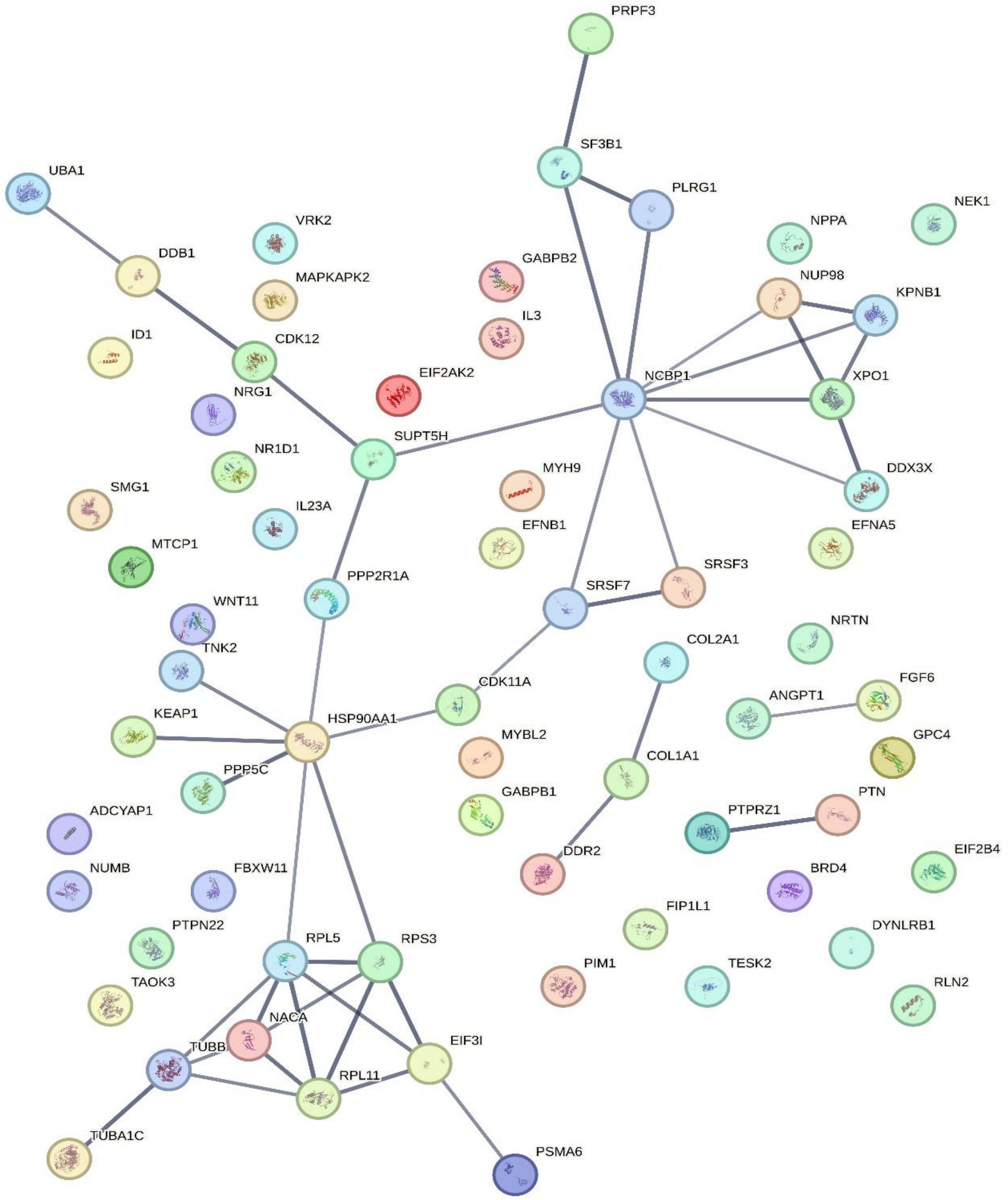

Figure S.6: PPI network between *68* unique driver proteins based on the STRING database by considering physical and functional connections with a confidence score of at least *0.7*.

NCBP1 Gene:

The NCBP1 gene is essential for cell growth and viability, playing a critical role in various cellular processes. It is involved in the formation of the HIV elongation complex and the transportation of mature mRNA, particularly in the absence of HIV Tat.

HSP90AA1 Gene:

The HSP90AA1 gene plays a crucial role in the drug-mediated inhibition of ERBB2 signaling. As a molecular chaperone, it aids in the maturation, structural maintenance, and regulation of specific target proteins essential for cell cycle control and signal transduction.

RPS3 Gene:

The ribosomal protein encoded by the RPS3 gene is a component of the 40S subunit and plays a significant role in translation initiation. This protein is upregulated in colon adenocarcinomas and adenomatous polyps and contributes to DNA damage repair and apoptosis through its endonuclease activity and involvement in CASP8 activation.

RPL5 and RPL11 Genes:

The proteins encoded by the RPL5 and RPL11 genes are associated with Diamond-Blackfan Anemia and may have tumor-suppressive effects. They activate downstream tumor suppressors and downregulate oncoprotein expression.

Table S.5: Table listing drugs that target key hub proteins identified in the PPI network analysis. Each drug's mechanism of action is detailed, highlighting its therapeutic relevance. Notably, the table includes drugs with established roles in cancer treatment, such as Doxorubicin hydrochloride and Dorlimomab aritox, as well as Obefazimod, which presents potential for drug repurposing in cancer therapy.

**Gene**	**Drug**	**Description**
NCBP1	Obefazimod	Obefazimod (ABX464) is a potent anti-HIV agent. Obefazimod inhibits HIV-1 replication in stimulated peripheral blood mononuclear cells (PBMCs) with an IC50 ranging between 0.1 μM and 0.5 μM
HSP90AA1	Doxorubicin hydrochloride	Doxorubicin (Hydroxydaunorubicin) hydrochloride, a cytotoxic anthracycline antibiotic, is an anti-cancer chemotherapy agent. Doxorubicin hydrochloride is a potent human DNA topoisomerase I and topoisomerase II inhibitor with IC50s of 0.8 μM and 2.67 μM, respectively. Doxorubicin hydrochloride reduces basal phosphorylation of AMPK and its downstream target acetyl-CoA carboxylase. Doxorubicin hydrochloride induces apoptosis and autophagy
RPS3	Dorlimomab aritox	Dorlimomab aritox (4197X-RA; MDX-RA (ricin A chain) immunotoxin) is a mouse-derived monoclonal antibody conjugated to ricin A.
RPL5 & RPL11	Exaluren	Exaluren (ELX-02) is a synthetic eukaryotic ribosome-selective glycoside that induces read through of nonsense mutations, resulting in normally localized full-length functional proteins. Exaluren is used for the research of cystic fibrosis caused by nonsense mutations.
